# Sex Dependence of Cognitive Functions in Bipolar Disorder

**DOI:** 10.1155/2014/418432

**Published:** 2014-01-29

**Authors:** Aleksandra Suwalska, Dorota Łojko

**Affiliations:** Department of Adult Psychiatry, University of Medical Sciences, 60-572 Poznań, Poland

## Abstract

The objective of the present study was to assess the performance of lithium treated euthymic bipolar patients in tests measuring spatial working memory (SWM), planning, and verbal fluency and to delineate the influence of gender on cognitive functioning. Fifty-nine euthymic bipolar patients, treated with lithium carbonate for at least 5 yr, were studied. Patients and controls underwent a neuropsychological assessment. Bipolar patients had significantly worse results than the healthy controls in the spatial memory and planning as well as verbal fluency tests. We detected a gender-related imbalance in the SWM results. Deficits in SWM were observed in male-only comparisons but not in female-only comparisons. The SWM scores were significantly poorer in male patients than in male controls. In female-only comparisons, female patients did not have significantly poorer SWM results in any category than their controls. Bipolar women scored worse in some other tests. The present study points to the different patterns of neuropsychological disturbances in female and male patients and suggests that sex-dependent differences should be taken into account in order to tailor the therapeutic intervention aimed at the improvement of cognitive functions.

## 1. Introduction

Bipolar disorder (BD) is a chronic and impairing mental disorder with estimated lifetime prevalence in general population of around 2.0% : 1.0% for BD type I (with a history of mania) and 1.1% for BD type II (with a history of hypomanic episodes only) [1, 2]. Bipolar disorder has been recognized as one of the most significant causes of increased mortality and morbidity due to mental illnesses [3]. The symptomatology of BD involves affective disturbances, cognitive deficits, and high rates of somatic and psychiatric comorbidity [4]. BD is a complex and heterogeneous illness.

The results of the studies investigating the prognostic role of gender in BD are inconsistent [1]. The majority of studies have reported equal prevalence of bipolar disorder (BD) in men and women, whilst bipolar II disorder is more frequent among females [5, 6]. Most studies report an increased risk of rapid cycling and mixed episodes in women [6]. Men usually present with manic episodes and have comorbid drug abuse, while women usually present with major depressive episode [7]. However, some authors have reported that the number of affective episodes of any polarity does not differ across gender [5]. Whereas some authors have noticed a later onset of bipolar disorder in women than in men [7], others have not noticed such a difference [5].

Women are more prone to experience mixed or dysphoric episodes and to present with seasonal pattern and rapid cycling [8] and tend to experience atypical depressive symptoms such as weight gain and hypersomnia [9]. Bipolar women more frequently have comorbid somatic illnesses, particularly thyroid diseases, migraine, and obesity [9], and tend to have more comorbid eating disorders, anxiety disorders [7], and bulimia [10], but lower rates of comorbid alcohol and cannabis abuse/dependence [9]. Female gender is associated not only with better medication adherence in bipolar disorder [9, 11, 12], but also with treatment refractory depression [9].

An association between bipolar disorder and cognitive impairment has repeatedly been described, even for euthymic patients [13]. Nowadays, the old Kraepelinian idea that bipolar patients, unlike those with schizophrenia, do not experience cognitive decline is known not to be true. Cognitive impairment, and especially executive dysfunction, has been recognized in BD patients. They experience neuropsychological disturbances during manic and depressive episodes and during remission periods [14–16]. Several metaanalyses [17–23] have provided evidence for trait deficits in verbal memory, and learning, executive functions, working memory, and response inhibition in remitted bipolar patients. The persistence of poor performance in these tasks in euthymic adult patients and in their unaffected relatives suggests that these specific cognitive impairments may be BD endophenotypes [24–26]. In healthy population, sexual dimorphism in cognitive functioning is well known and widely described. The classical dichotomy claims that men are better at spatial abilities and women at verbal skills.

Some studies investigating sex differences in spatial working memory have recently brought equivocal results [27] with men outperforming women only as regards the visuospatial component of the spatial rotation test. Men performed more quickly and with a greater accuracy in navigation tests [28, 29]. Several studies indicate that in tasks involving remembering objects in an array, women outperform men. A female advantage is described in tasks of verbal memory, episodic and autobiographical memory, some tasks involving processing speed and emotional memory [27]. Female patients are superior to male patients in verbal memory and fluency tests, both phonological and semantic [27, 30]. Several studies indicate sexual differences in processes of working memory [31]. In addition to working memory test results, male advantage in the Stocking of Cambridge [32] test has been reported, suggesting sex differences in the planning component of executive function [33]. Several studies have shown faster psychomotor speed in men [34] and poorer attention and psychomotor performance in women [35].

As brain tissue and neurons are neurobiological substrates for cognitive processes, it is unsurprising that healthy population has sexually dimorphic brain in morphology and physiology. The nature of this difference is unclear [36–38]. Differences between male and female gray matter concentration [39] or cortical thickness [40] as well as callosal thickness [41] were described. Male brain is of a larger volume (109–112% of female brain); males have a greater volume of cerebrospinal fluid [42]. The total percentage of grey matter has been found to be smaller in men, and cortical gyrification and cortical complexity have been higher in women [43]. Sex differences include larger amygdala and hypothalamus in men and larger caudate and hippocampus in women [44, 45]. Positron emission tomography (PET) and SPECT (single photon emission computed tomography) have revealed sex-dependent differences in the central dopaminergic, serotonergic, and gamma-aminobutyric acid (GABA) ergic function [45]. Enhanced dopaminergic function in women has been reported [45], and dopamine is involved in prefrontal or executive tasks as many dopamine receptors are located in the frontal areas of the brain [46].

Although cognitive abnormalities are a well established feature of BD, and the sexual dimorphism of the healthy brain is well known, there is limited information regarding whether gender may influence the pattern and severity of cognitive impairment in BD. Only a few studies have directly addressed the issue of gender differences in cognition of bipolar patients [47, 48].

### 1.1. Objective

The objective of the present study was to assess the performance of lithium treated euthymic bipolar patients in tests measuring spatial working memory and planning as well as verbal fluency and to delineate the influence of gender on the cognitive functioning of patients.

## 2. Methods

Fifty-nine patients (24 male, 35 female), aged 26–75 yr (mean ± S.D.: 52 ± 10 yr) with bipolar disorder attending the outpatient lithium clinic at the Department of Psychiatry, Poznan University of Medical Sciences, were studied. A consensus diagnosis by two psychiatrists was made for each patient, according to the DSM-IV criteria (structured clinical interview for DSM-IV Axis I-SCID) [49].

The patients were treated with lithium carbonate for at least 5 yr (5–27 yr, mean 13 yr). Serum concentration of lithium was maintained in the range between 0.5 and 0.8 mmol/L. The course of the illness was assessed retrospectively, based on the analysis of medical outpatient charts, in inpatient records, and semistructured interviews. The patients had variable numbers of episodes during lithium therapy (0–30, mean 5 ± 6). Among patients, 13 were excellent lithium responders, defined as having had no affective episodes while on lithium monotherapy for the entire period of lithium administration [50]. The proportion of the excellent lithium responders in male and female groups did not differ significantly.

On the day of study, all patients were euthymic, as defined by a score of <7 on the 17-item Hamilton Depression Rating Scale (HAMD17) [51] and a score of <7 on the Young Mania Rating Scale (YMRS) [52]. Fifty-nine healthy controls without personal or family history of mental illness recruited from the local community were matched head-to-head by age, gender, and education level. The study was approved by the Ethics Committee of Poznan University of Medical Sciences. Patients and volunteers gave their written informed consent after hearing a complete description of the study.

The exclusion criteria for both groups included dementia symptoms, a history of head trauma with loss of consciousness, substance abuse, epilepsy, and severe somatic illnesses.

Patients and controls underwent an extensive neuropsychological assessment. Several “paper-and-pencil” tests, The Trail Making Test (TMT) [53] and Verbal Fluency Test [54] as well as selected tests from the Cambridge Automated Neuropsychological Test Battery (CANTAB; CeNeS Ltd., Cambridge, UK)—Spatial Working Memory (SWM), spatial span (SSP), and Stocking of Cambridge [55–58]—were employed.

The tests were described in detail in our previous paper [14]. The* Trail Making Test (TMT) *consists of two parts. TMT requires subjects to connect 25 consecutively numbered circles (part A) and 25 numbered and lettered circles by shifting between the two sets (part B) as quickly as possible; the test is very sensitive to cerebral dysfunction. Part A of the test measures psychomotor speed; the results of part B reflect the ability to shift strategy and assess executive function and visuospatial working memory [59]. Time is recorded in seconds.

*Verbal Fluency Tests. *Phonologic verbal fluency was studied by asking subjects to generate as many words as possible that begin with each of the letters F, A, and S, in consecutive 1 min time periods (FAS Test, from the COWAT: Controlled Oral Word Association Test) [60]. Semantic verbal fluency was measured with the Category Instant Generation Test, by naming as many items as possible in a given category (animals, vegetables, and fruit) within the same time limit. Scores were the sum of all acceptable words produced in the three trials. Verbal fluency is a sensitive measure of executive functions, as it requires the subject to generate their own strategy [61].

*Spatial Working Memory (SWM).* It is a test of the subject's ability to retain spatial information and to manipulate the remembered items in the working memory. The test measures the working memory for spatial stimuli and requires the subject to use mnemonic information to work towards a goal. Subjects are required to search through boxes that appear on the screen with the aim of finding the “blue tokens” hidden inside. The outcome measures for SWM include errors and strategy; higher strategy scores represent lower use of a strategy. This test is used to detect frontal lobe and executive dysfunction.

*Spatial Span (SSP). * A visuospatial analogue of the Digit Span test assesses working memory capacity. The Spatial Span is calculated at the highest level at which the subject successfully remembers at least one sequence of boxes.

*CANTAB Stockings of Cambridge Planning Task [32].* This is a visuospatial planning test based on the Tower of London task [62]. The participant uses the balls in the lower display to copy the pattern shown in the upper display. It is the test of spatial planning ability and a measure of frontal lobe function [33]. Screenshots and detailed overviews of the tests are available online [63].

## 3. Statistics and Results 

Statistical analyses were carried out with Statistica version 10.0 for Windows. To evaluate the normality of distribution of the variables, the Shapiro-Wilk test was applied. As most of the investigated variables were not normally distributed, nonparametric tests were employed. Between-group differences in the demographic characteristics and neuropsychological tests were assessed by the Mann-Whitney test. All the results were expressed as the mean and standard deviation (S.D.). Statistical significance was set at *P* < 0.05 for all analyses.

Patients and controls were well matched for sex, age, and level of education. Patients' HDRS score (2.5 ± 1.8) was low but it was higher than controls (0.7 ± 1.1) with *P* < 0.01. No differences between controls and patients in the YMRS scale were present. There were no significant differences between female patients and male patients as regards the age of illness onset, duration of illness, number of phases, or the duration of lithium treatment. Male and female patients had no significant differences regarding HDRS scores or YMRS scores on the day of the study. Male controls were younger than female controls (*P* < 0.05) (Table 1).

Bipolar patients had significantly worse results than the healthy controls (Table 2) on the Spatial Span, Spatial Working Memory, Stockings of Cambridge, Trail Making Test, and verbal fluency tests. As far as the SWM strategy scores and SWM between errors scores are concerned, the diagnostic group performed worse; however no statistical difference was detected as regards the SWM within errors score. The number of errors in SSP did not differ between the groups.

Gender effect (Tables 3, 4, and 5). In the bipolar group, the results of women and men were almost identical. Bipolar male patients performed worse on the semantic fluency test (*P* < 0.05) but scored better on the SOC problems solved in minimum moves (*P* < 0.01), which assesses planning ability. In the control group, male individuals scored significantly better on TMT A, made significantly less between-search errors in SWM (*P* < 0.05), and tended to have a better strategy in SWM than female controls (*P* = 0.6). No more differences between the healthy subjects were found. We detected a gender-related imbalance in the SWM results. Deficits in SWM were observed in male-only comparisons (both in the Strategy and Between-errors scores) but not in female-only comparisons. The SWM Strategy scores were significantly poorer in male patients than in male controls (*P* < 0.05). Male patients also made significantly more between-search errors (*P* < 0.01). In the SWM within errors scores a trend towards worse results in bipolar men was detected as well (*P* = 0.09). In female-only comparisons, female patients did not have significantly poorer SWM results in any category (strategy scores, between-search errors, and within errors) than their controls. Bipolar women scored worse in SSP length, SOC mean initial thinking time, TMT-both parts, and the semantic fluency test.

## 4. Discussion

Over the last decade, a growing body of evidence has shown the persistence of cognitive deficits during all phases of bipolar disorder, including the affective states and the remission (euthymia) [17, 23]. Euthymic BD patients have been found to suffer from marked impairment across the cognitive domains of attention/processing speed, verbal learning/memory, and executive functioning in many studies [64, 65].

The results of our study point to the deficits in visuospatial abilities in the group of euthymic bipolar patients. The Spatial Working Memory (SWM) test assesses subject's ability to retain and manipulate spatial information. Bipolar patients in our study made more between-search errors and had poorer strategy scores in that test. Similar results of spatial and verbal memory impairments in BD have been reported by Yatham et al. [65] but not by Pirkola et al. [25]. The capacity of visuospatial working memory was worse in the patients than in the healthy controls as the Spatial Span length was significantly shorter in the diagnostic group. Visuospatial planning was assessed using the SOC test. The results of SOC subsequent thinking time and SOC problems solved in minimum moves did not differ between the groups whilst the mean initial thinking time of the SOC test was significantly longer in the patients' group. Worse results in the TMT part B also showed deficits in executive functions and visuospatial working memory in bipolar patients. TMT has been recently described as a robust measure of cognitive impairments in bipolar disorder patients [13]. 

These findings coincide with the existing literature, as specific deficits in working memory and executive control have been identified in euthymic bipolar patients [17, 66, 67].

Moreover, our patients also had worse results in the FAS test reflecting the impaired executive function, which are consistent with the results of BD samples in metaanalysis by Kurtz and Gerraty [23].

The precise etiology and pathophysiology of cognitive impairment in BD have not been fully elucidated. During working memory tasks dorsolateral prefrontal cortex (DLPFC), inferior prefrontal cortex, areas of the parietal lobe, and the anterior cingulate cortex are involved [68, 69]. There is a growing body of evidence implying that the ventral frontopolar dysfunction is a core abnormality in BD [70]. Impairments of spatial working memory in schizophrenic patients were well described and imply the frontal-striatal-parietal (particularly prefrontal cortex) neural networks dysfunction [71].

In our patients' group, the deficits of visuospatial memory in SWM were more prominent in male patients. Whereas the results of visuospatial tests of healthy men were significantly better than those of control women on SWM between errors and they tended to be better on the SWM strategy, no such difference was observed between male and female BD patients in the within-patients comparison.

Gender-related disequilibrium in SWM reported in healthy individuals [23] was absent in our bipolar patients. In bipolar male patients, no correlation between the SWM results and clinical factors was observed. Female patients' SWM results correlated with the duration of illness, and such a correlation suggests that some impairments may track illness progression [13].

According to Barrett's hypothesis, this suggests that the impact of BD on SWM is different depending on patients' gender [72]. The authors [72] have compared the results of 12 male and 14 female BD outpatients with those of healthy controls on letter fluency, spatial working memory, planning, and cognitive set shifting. Male patients have performed worse than female patients in measures of spatial working memory, indicative of poor retention of the visuospatial information. However, male patients in that study had been both older and more symptomatic than female patients.

Carrus et al. [47] have evaluated the performance of 86 remitted patients with BD type I (36 male and 50 female) and 46 healthy participants (21 male and 25 female) on tasks of general intellectual ability, memory encoding, recognition and retrieval, response inhibition, and executive function (abstraction and perseveration). They have found a gender effect and an interaction between diagnosis and gender on immediate memory, both auditory and visual, but not on general intellectual ability, concept formation, and perseveration or response inhibition. Male patients have performed worse compared with female patients and healthy controls. Immediate memory has correlated with GAF scores and this association has been statistically significant for male patients [47].

In another study, a group of 106 BD I patients (55 women/51 men) has been assessed with an extensive neuropsychological test battery and the social functioning questionnaire and compared with the control group and schizophrenic patients group [73]. Clinical groups have performed worse than healthy controls for all neuropsychological tests (except attention). The authors have found neurocognitive sex differences for bipolar disorder but their data have not supported the hypothesis of disruption of normal sexual dimorphisms in BD patients, which has been reported for schizophrenic patients. In terms of cognitive results, there has been no general “different sex difference” between bipolar disorder patients compared with healthy controls. According to Vaskinn et al. [73] the neurocognitive sex differences found in their study could depend on the normal sexual dimorphisms in brain structures seen in healthy populations.

Greater impairment in working memory including spatial memory has been observed in bipolar patients with a history of psychosis in comparison with the remaining bipolar patients [74].

The results of the study of cognitive functions of 60 euthymic patients who had recently experienced their first manic or mixed episode [48] have been consistent with those of Vaskinn et al. [73] but somewhat disagreed with the results of Barrett et al. [72] and Carrus et al. [47]. Popuri [48] has found that bipolar patients as a group showing a poorer cognitive performance than age- and sex-matched healthy controls. Sex has been an important determinant of the neurocognitive function: males performed better than females on measures of sustained attention and set shifting, whereas females have tended to perform better than males in verbal learning. There have been no group and sex interactions indicating that sex has the same impact on the neurocognitive function in bipolar I patients as in healthy controls. Data concerning gender differences in brain morphology are also sparse [70]. Mackay et al. [75] have reported that the normal sex difference in brain asymmetry tends to be diminished in patients with bipolar disorder. Bipolar disorder has been characterized by an increased cerebrospinal fluid volume, and although there has been no overall change in brain volume, male patients had increased and female patients decreased lobe volumes relative to sex-matched controls. Considered as a neurodevelopmental illness, bipolar disorder may represent a sex-dependent anomaly in the growth of the cerebral cortex [75]. In the review of neuroimaging findings in BD patients, Jogia et al. [70] suggest that the sex of an individual modulates the structure and function within the subcortical and cortical regions implicated in the disease expression. The influence of sex on structural brain morphology may probably be greater during the development than later in life, and it is unknown how prescribed medicines may influence both the morphology and functioning of brain. Persistent cognitive deficits have a number of clinical implications for BD patients. Intact cognitive functioning is critical for success in independent, useful functioning in everyday life [54]. Impaired executive functions have been found to relate to poor outcome and problematical management in brain injury [76] and poor psychosocial and occupational functioning in BD patients [77]. In this way, gender might be a factor associated with poorer social outcome in BD [6]. One of the most important aims of future research should therefore be the identification of the underlying neurobiology of neurocognitive impairment in euthymic patients, thereby, providing a target for therapeutic intervention.

Many studies have shown that BD patients have cognitive executive-type dysfunctions [67, 77, 78] which may potentially serve as endophenotypes for BD. As many authors underline, affective symptoms (clinical phenotype) can probably contribute less to the knowledge of the underlying pathology of BD than neurocognitive deficits [79]. Metaanalyses and critical literature reviews [18, 19, 21, 23] based on numerous studies suggest that cognitive performance is something like cover measures for BD traits, that is, measures between the underlying genetic contribution and easily seen symptoms [79]. The metaanalysis of neuropsychological functioning in euthymic bipolar disorder conducted by Mann-Wrobel et al. [17] has concluded that cognitive impairments in euthymic bipolar disorders are rather generalized than specific. On the other hand, age, illness duration, education, and clinical course may moderate these broad cognitive effects. The direction of impact is unknown; causality cannot be determined from correlation analyses; [66] patients with cognitive impairment may develop a more severe type and course of BD, and a severe BD course leads to a greater impairment in cognitive functions and, consequently, a poorer functional outcome.

Cognitive and psychoeducational rehabilitation programs may be warranted to improve the long-term outcome for some patients [66]. They should be adjusted to the individual needs, and patients' gender has to be taken into account in terms of different gender-related cognitive impairment.

We are aware of the limitations of this study, including a possible impact of medications. A comparison between our results and those of other studies is biased by different inclusion criteria, and different stage of illness, treatment methods, and differences in somatic comorbidity (and comedication) [20]. All our patients were on prophylactic lithium treatment (mean 12.3 yr) just like those referred to in Barrett's paper [72]. Lithium has been used for over 50 years as a recognized medication for the treatment of both mania and depression, and the maintenance of well-being in bipolar disorder [3]. Lithium is an efficacious agent for the treatment of bipolar disorder, but it is unclear to what extent its long-term use may result in neuroprotective or neurotoxic consequences [80]. In the last two decades, a growing body of evidence has shown that lithium has several neuroprotective effects [81]. Neuroimaging studies in humans have demonstrated that chronic use is associated with cortical thickening, higher volume of the hippocampus and amygdala, and neuronal viability in bipolar patients on lithium treatment. Chronic lithium intake is associated with a reduced risk of Alzheimer's disease in subjects with bipolar disorder [81]. On the other hand, lithium has been reported to be associated with impairments in learning, memory, and psychomotor performance [82], but this association may be mediated by other factors, such as other medications or symptoms of the affective phase [26]. The authors of another review [83] have concluded that lithium carbonate has only mild negative effects on immediate verbal learning and memory and creativity and a moderate adverse effect on psychomotor performance in euthymic patients with affective disorders. Based on the review of literature, Fountoulakis et al. [80] have concluded that although more papers are in favour of the toxic effect, the great difference in the type of papers that support either hypothesis, along with publication bias and methodological issues, makes conclusions difficult.

## 5. Conclusion

The results of our study point to the presence of cognitive deficits in euthymic bipolar patients; therefore they support the statement of Bowden and Singh [84] pointing to the difference between remission and response and between syndromal recovery (amelioration of clinical symptoms) and functional recovery. Our results substantiate the postulation of Goodwin et al. [85] that neurocognitive impairment should be considered as a potential therapeutic target, to improve cognitive functions and the functional outcome of bipolar patients. Even though further studies are needed to clarify the relationship between gender and cognitive functions in bipolar disorder, the present study points to the different patterns of neuropsychological disturbances in female and male patients and suggests that sex-dependent differences should be taken into account in order to tailor the therapeutic intervention aimed at the improvement of cognitive functions.

## Figures and Tables

**Table 1:** Sociodemographic and clinical characteristics (values expressed as means and standard deviations shown in brackets).

	Patients group			Control group		
	Overall (*n* = 59)	Males (*n* = 24)	Females (*n* = 35)	Overall (*n* = 59)	Males (*n* = 24)	Females (*n* = 35)
Age (years)	52.4 (10.2)	50.0 (10.0)	53.9 (10.2)	52.7 (11.7)	48.5 (10.6)	55.6 (11.6)^a^
Education (years)	13.7 (3.6)	14.3 (3.6)	13.4 (3.5)	13.3 (2.5)	13.6 (1.8)	13.1 (2.8)
Age at illness onset (years)	30.1 (10.8)	28.4 (10.4)	31.7 (11.1)	—	—	—
Number of episodes	13.3 (8.2)	13.3 (8.7)	13.2 (7.8)	—	—	—
Duration of illness (years)	21.8 (10.7)	21.4 (10.6)	22.2 (11.0)	—	—	—
Duration of lithium treatment (years)	12.3 (8.5)	11.8 (7.4)	12.6 (9.4)	—	—	—
HDRS	2.5 (1.8)	2.5 (1.7)	2.6 (2.1)	0.7 (1.1)^b^	0.3 (0.5)	1 (1.4)
YMRS	0.6 (0.9)	0.7 (1.0)	0.3 (0.7)	0.4 (0.7)	0.3 (0.8)	0.4 (0.7)

YMRS: Young Mania Rating Scale; HDRS: Hamilton Depression Rating Scale.

^a^ *P* < 0.05: difference between male controls and female controls.

^b^ *P* < 0.01: difference between patients and control group.

**Table 2:** Group differences in cognitive performance (values expressed as means and standard deviations shown in brackets).

Test	Patients	Controls	Statistics
SSP span length	4.9 (1.1)	5.4 (1.1)	*P* < 0.01
SSP total errors	12.6 (5.2)	12.1 (5.3)	NS
SWM strategy	37.3 (4.3)	34.9 (5.1)	*P* < 0.05
SWM within errors	4.8 (5.4)	3.4 (4.2)	NS
SWM between errors	46.5 (19.8)	35.1 (19.8)	*P* < 0.01
SOC mean initial thinking time (5 moves)	11497.6 (11.849.0)	8816.8 (5717.0)	*P* < 0.01
SOC mean subsequent thinking time (5 moves)	3636.1 (2472.2)	3270.0 (3323.9)	NS
SOC problems solved in minimum moves	7.6 (1.6)	7.5 (1.7)	NS
Semantic fluency (number of words)	41.2 (8.6)	49.1 (8.9)	*P* < 0.001
Phonemic fluency (number of words)	28.7 (8.7)	36.4 (14.6)	*P* < 0.01
TMT A (time (sec))	44.4 (17.5)	34.9 (10)	*P* < 0.01
TMT B (time (sec))	113.9 (46.8)	81.9 (43.6)	*P* < 0.001

**Table 3:** Within-group comparisons of cognitive functions (values expressed as means and standard deviations shown in brackets).

	Controls			Patients		
	Males	Females	*P *	Males	Females	*P *
SSP span length	5.6 (1.3)	5.4 (1.0)	NS	5.0 (1.3)	4.9 (0.9)	NS
SSP total errors	12.0 (6.2)	12.1 (4.7)	NS	14.4 (6.9)	11.7 (4.1)	NS
SWM strategy	33.4 (5.8)	35.9 (4.4)	*P* < 0.05	37. 0 (5.6)	37.5 (3.3)	NS
SWM within errors	2.6 (4.3)	4.1 (4.1)	*P* < 0.05	6.7 (7.9)	3.6 (2.74)	NS
SWM between errors	28.4 (19.7)	39.8 (18.8)	*P* < 0.05	45.8 (18.7)	47.0 (20.7)	NS
SOC mean initial thinking time (5 moves)	7969.6 (6475.0)	6059.2 (5116.2)	NS	9248.9 (5951.1)	13039.6 (14475.1)	NS
SOC mean subsequent thinking time (5 moves)	2308.5 (2168.9)	3901.9 (3800.2)	NS	2935.7 (2152.5)	4116.3 (2590.0)	NS
SOC problems solved in minimum moves	7.7 (1.48)	7.4 (1.8)	NS	8.3 (1.5)	7.1 (1.5)	*P* < 0.01
Semantic fluency (number of words)	49.2 (9.7)	49.1 (8.4)	NS	37.6 (5.8)	43.2 (9.3)	*P* < 0.05
Phonemic fluency (number of words)	37.5 (12.9)	35.7 (15.8)	NS	26.9 (8.3)	29.7 (8.9)	NS
TMT A (time (sec))	30.9 (7.1)	37.3 (10.7)	*P* < 0.05	43.1 (17.8)	45.1 (17.5)	NS
TMT B (time (sec))	71.6 (16.9)	88.0 (52.9)	NS	115.7 (46.6)	112.9 (47.6)	NS

**Table 4:** Male-only and female-only comparisons of cognitive functions (values expressed as means and standard deviations shown in brackets).

	Males			Females		
	Patients	Controls	*P *	Patients	Controls	*P *
SSP span length	5.0 (1.3)	5.6 (1.3)	NS	4.9 (0.9)	5.4 (1.0)	*P* < 0.05
SSP total errors	14.4 (6.9)	12.0 (6.2)	NS	11.7 (4.1)	12.1 (4.7)	NS
SWM strategy	37.0 (5.6)	33.5 (5.8)	*P* < 0.05	37.5 (3.3)	36.0 (4.4)	NS
SWM within errors	6.7 (8.0)	2.6 (4.3)	NS	3.6 (2.7)	4.1 (4.1)	NS
SWM between errors	45.8 (18.7)	28.5 (19.7)	*P* < 0.01	47.0 (20.7)	39.8 (18.8)	NS
SOC mean initial thinking time (5 moves)	9248.9 (5951.1)	7969.6 (6475.0)	NS	13039.6 (14475.1)	6059.2 (5116.2)	*P* < 0.01
SOC mean subsequent thinking time (5 moves)	2935.7 (2152.5)	2308.5 (2168.9)	NS	4116.3 (2590.0)	3901.9 (3800.2)	NS
SOC problems solved in minimum moves	8.3 (1.5)	7.7 (1.5)	NS	7.1 (1.5)	7.4 (1.8)	NS
Semantic fluency (number of words)	37.6 (5.8)	49.2 (9.7)	*P* < 0.001	43.2 (9.3)	49.1 (8.4)	*P* < 0.01
Phonemic fluency (number of words) *P* < 0.004	26.9 (8.3)	37.5 (12.9)	*P* < 0.01	29.7 (8.9)	35.7 (15.8)	NS
TMT A (time (sec))	43.1 (17.8)	30.9 (7.2)	*P* < 0.05	45.1 (17.5)	37.3 (10.7)	*P* < 0.05
TMT B (time (sec))	115.7 (46.6)	71.6 (16.9)	*P* < 0.001	112.9 (47.6)	88.0 (52.9)	*P* < 0.01

**Table 5:** Correlations between clinical factors and cognitive tests results (*significant at *P* < 0.05) in bipolar patients.

		Age at onset	Number of episodes	Illness duration	YMRS	HDRS
SSP span length	O	0.01	−0.44*	−0.29*	0.01	−0.19
	M	0.01	−0.40	−0.12	−0.01	−0.24
	F	0.04	−0.51*	−0.49*	0.18	−0.08

SWM strategy	O	−0.09	0.20	0.25	0.16	0.14
	M	−0.00	0.15	0.05	0.25	−0.06
	F	−0.26	0.28	0.48*	0.04	0.46

SWM between errors	O	−0.00	0.24	0.35*	0.02	−0.18
	M	−0.02	0.16	0.02	0.12	−0.12
	F	−0.04	0.31	0.58*	−0.15	−0.26

SOC mean initial thinking time (5 moves)	O	0.23	0.21	−0.19	0.32	0.42*
	M	−0.31	0.58*	0.48*	0.22	0.32
	F	0.43*	0.08	−0.46*	0.93*	0.60

SOC mean subsequent thinking time (5 moves)	O	0.17	−0.00	−0.05	0.25	−0.18
	M	0.04	0.32	−0.01	0.37	−0.14
	F	0.19	−0.23	−0.10	0.24	−0.35

SOC problems solved in minimum moves	O	0.33*	−0.28*	−0.16	0.24	0.12
	M	0.36	−0.50*	−0.15	0.19	0.11
	F	0.46*	−0.11	−0.17	0.32	0.27

Semantic fluency	O	−0.14	−0.05	−0.18	−0.13	0.21
	M	−0.45	0.02	−0.03	−0.03	0.18
	F	−0.06	−0.07	−0.28	−0.12	0.34

Phonemic fluency	O	−0.03	−0.27	−0.26	0.09	0.06
	M	−0.13	−0.19	0.02	0.09	−0.09
	F	0.01	−0.31	−0.43*	0.32	0.52

TMT A	O	0.25	0.07	0.16	0.05	−0.18
	M	0.37	0.30	0.03	0.26	0.11
	F	0.15	−0.10	0.24	−0.68*	−0.80*

TMT B	O	0.20	0.21	0.05	0.20	−0.21
	M	0.30	0.11	−0.37	0.35	−0.02
	F	0.14	0.27	0.26	−0.28	−0.53

O: overall group of bipolar patients.

M: male patients.

F: female patients.

YMRS: Young Mania Rating Scale.

HDRS: Hamilton Depression Rating Scale.
